# Primary lung hyalinizing clear cell carcinoma: a diagnostic challenge in biopsy

**DOI:** 10.1186/s13000-022-01216-5

**Published:** 2022-03-27

**Authors:** Yanling Zhang, Wen Han, Jun Zhou, Xiang Yong

**Affiliations:** 1 Department of Oncology, Anhui Wanbei Coal-Electricity Group General Hospital, Suzhou, China; 2 Department of Pathology, People’s Hospital of Xinjiang Uygur Autonomous Region, Xinjiang, China; 3 Department of Pathology, Affiliated Cancer Hospital of Zhengzhou University, Henan Cancer Hospital, Zhengzhou, 450003 China; 4 Department of Pathology, Anhui Wanbei Coal-Electricity Group General Hospital, Suzhou, 234000 People’s Republic of China

**Keywords:** Hyalinizing clear cell carcinoma, Biopsy, Lung, EWSR1-ATF1, Salivary gland tumors, Molecular pathology

## Abstract

**Introduction:**

Hyalinizing clear cell carcinomas (HCCCs) are rare, low-grade, malignant tumors. They most commonly involve the minor salivary glands of the head and neck. HCCC that occurs in uncommon locations and examining samples from small biopsy pose a diagnostic challenge for most pathologists.

**Case presentation:**

We herein report a primary pulmonary HCCC diagnosed by small biopsy and summarize its histologic, immunophenotypic, and molecular features along with a review of 11 previously reported cases to emphasize the potential diagnostic pitfalls.

**Conclusions:**

Small biopsy diagnosis of primary pulmonary HCCC is challenging. A collection of mimics needed to be ruled out. Awareness of the key morphologic features of pulmonary HCCC combined with essential immunohistochemistry and molecular tests contributes to the correct diagnosis.

## Introduction

Primary pulmonary salivary gland-type cancers are rare, accounting for under 1% of all lung tumors [1]. Along with mucoepidermoid carcinoma, adenoid cystic carcinoma, and epithelial-myoepithelial carcinoma, primary pulmonary hyalinizing clear cell carcinoma (HCCC), as a new entity, has been included in the 2021 WHO classification of pulmonary tumors [1].

The HCCC is a rare salivary gland tumor. It was first described in 1994 by Milchgrub et al. [2]. It most commonly involves the minor salivary glands of the head and neck, especially the palate and oral base [3]. When it occurs in uncommon locations, such as the bronchus or nasopharynx oropharynx, or when pathologists must examine a small biopsy sample, the diagnostic challenge can be considerable. To date, 11 primary pulmonary HCCC cases from eight articles have been reported [4–11]. However, only one case was diagnosed from a small biopsy [8]. Herein, we report an additional case of primary pulmonary HCCC diagnosed by small biopsy with an emphasis on the potential diagnostic pitfalls.

## Case presentation

### Clinical history

**Fig. 1:**
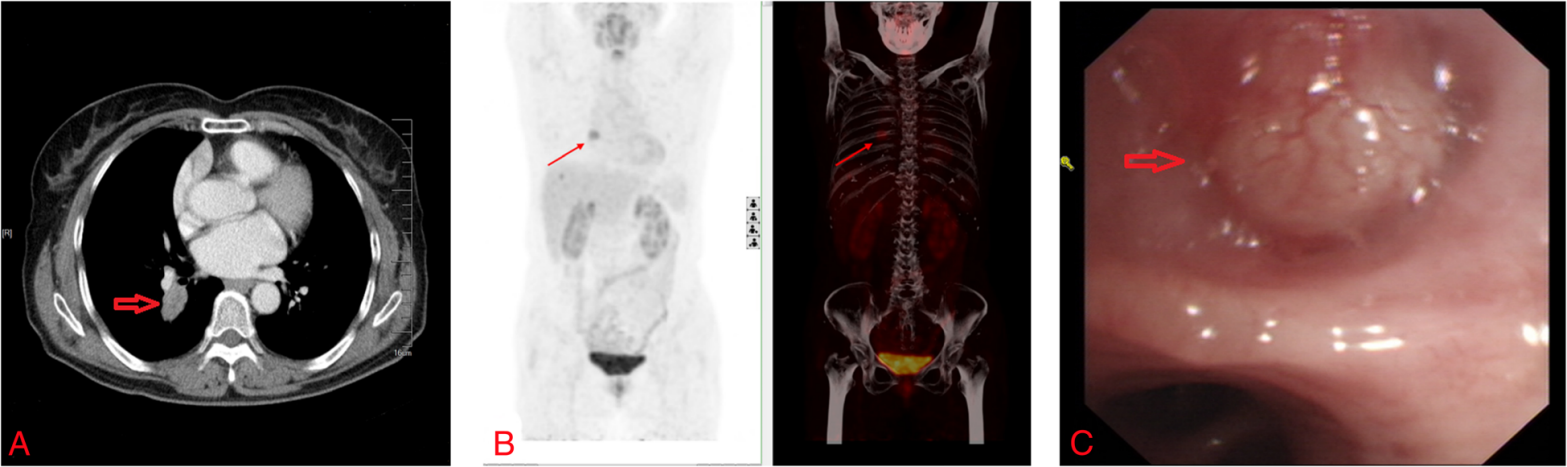
**A** Chest CT scan revealed an interval enlargement of a 2.8-cm dorsal segment of the right lower lobe mass (red arrow). **B** PET-CT showed central hypermetabolic activity only in the right lower lobe mass (red arrow). **C** Bronchoscopy revealed a dorsal segment of the right lower lobe with a possible cancerous mass (red arrow)

A 57-year-old Asian woman was admitted due to a finding of pulmonary mass in the right lower lobe upon physical examination. The patient, an ex-smoker (10 cigarettes a day × 20 years), had no specific medical history otherwise. The chest computerized tomography (CT) scan (Fig. 1A) revealed a 2.8-cm dorsal segment of the right lower lobe mass. A positron emission tomography scan (PET-CT) showed central hypermetabolic activity only in the right lower lobe, raising suspicions of a neoplastic process (Fig. 1B). Bronchoscopy revealed a dorsal segment of the right lower lobe mass (Fig. 1C), and a biopsy was performed.

### Histopathology and ancillary testing

**Fig. 2:**
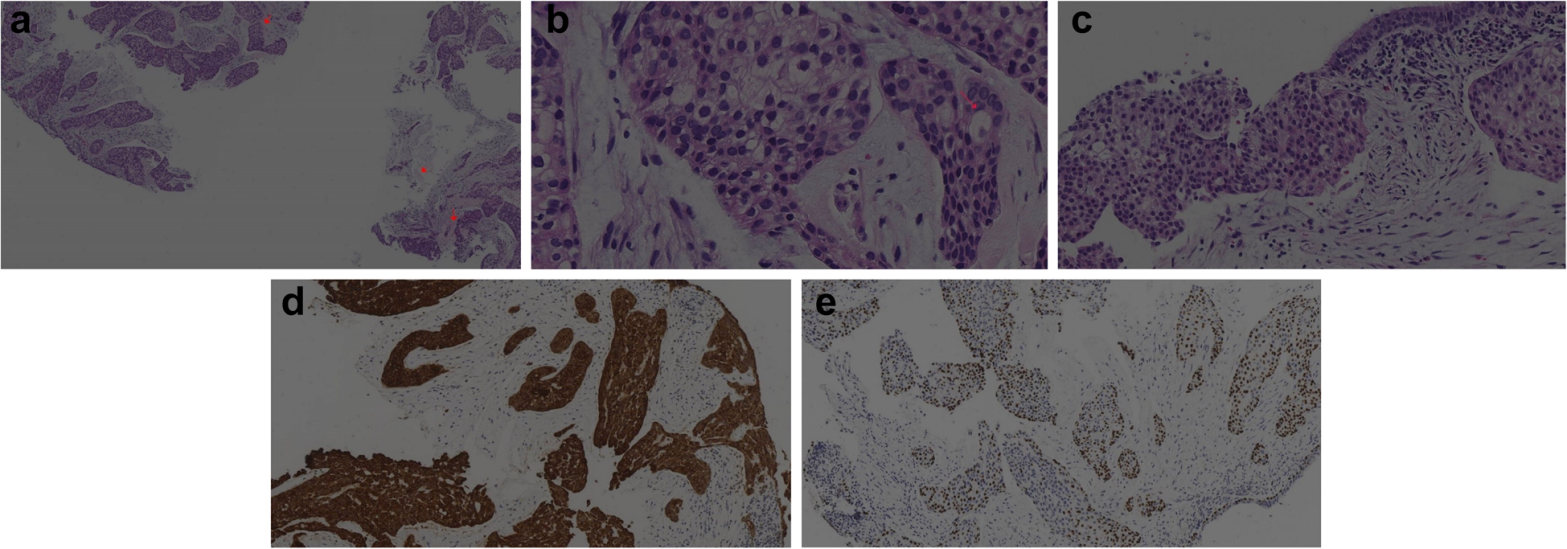
**A** The tumor cells arranged in irregular nests, cords, trabeculae, and tubular shapes with loose mucinous stroma and focal stroma hyalinized (red arrow) (HEx 40). **B** Atypical small-to-medium-sized epithelioid cells with light eosinophilic to clear cytoplasm lacking significant pleomorphism and mitotic activity arranged in irregular nests, cords, trabeculae, and tubular patterns (red arrow) with a loose myxoid stroma **(C)** (HEx 200). Focally, the tumor cells appeared to be connected to the basal layer of the bronchial epithelium. **D** Immunohistochemistry revealed that the tumor cells were positive for CK7. **E** Immunohistochemistry revealed that most of the tumor cells were positive for p40

**Fig. 3:**
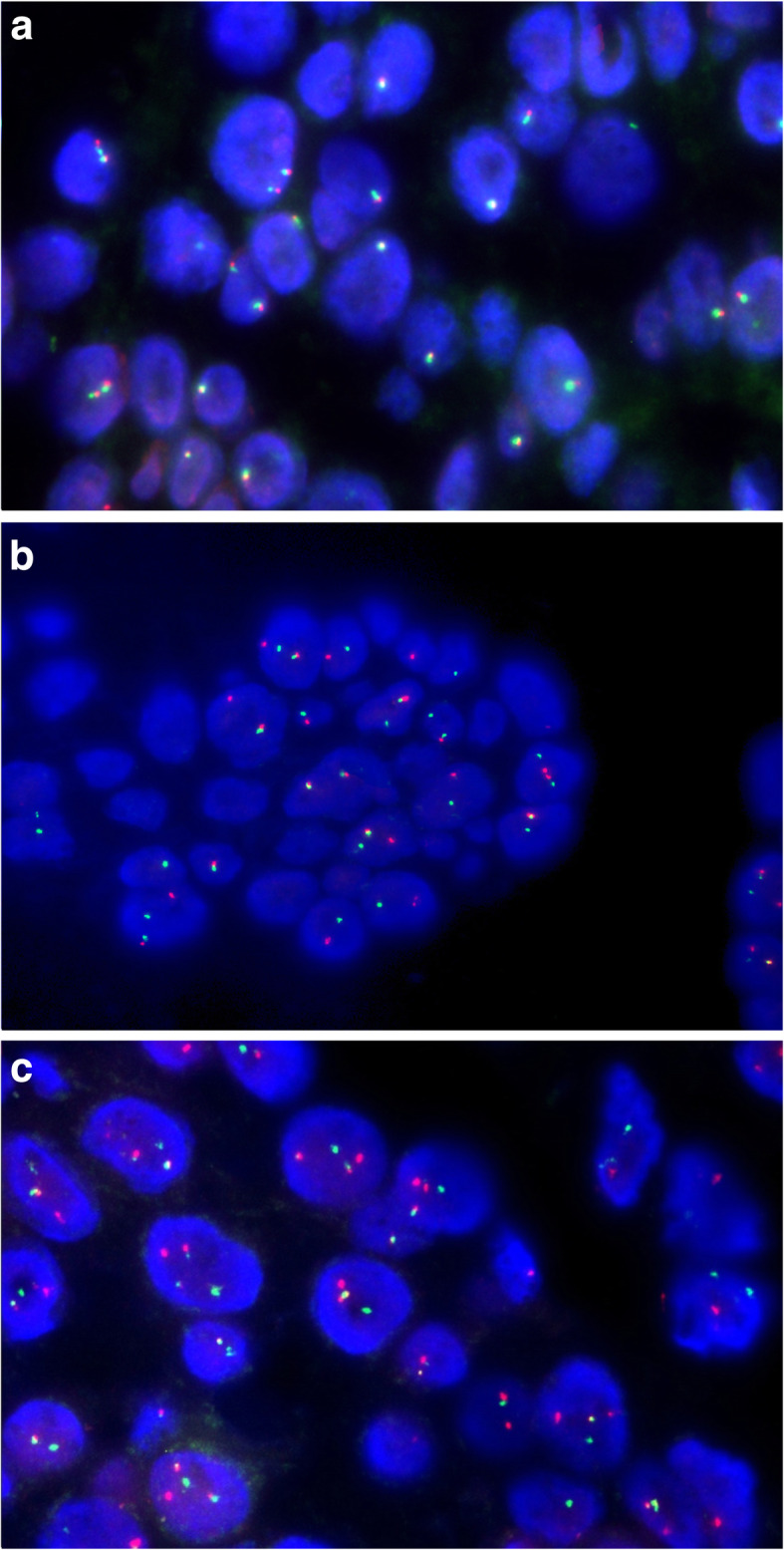
**A** Fluorescence in situ hybridization (FISH) was negative for MAML2 rearrangement (the MAML2 locus is rearranged in 2% of neoplastic cells as characterized by one green, one red, and one fusion signal). **B** Fluorescence in situ hybridization (FISH) was positive for EWSR1 gene rearrangement (the EWSR1 locus is rearranged in 25% of neoplastic cells as characterized by one green, one red, and one fusion signal). **C** Fluorescence in situ hybridization (FISH) was positive for EWSR1-ATF1 fusion (EWSR1-ATF1 fusion in 32% of neoplastic cells as characterized by one green, one red, and two fusion signal)

The hematoxylin and eosin (HE)–stained sections of the biopsy yielded a neoplasm composed of atypical small-to-medium sized epithelioid cells with light eosinophilic to clear cytoplasm and round-to-oval nuclei and inconspicuous nucleoli lacking significant pleomorphism and mitotic activity. The tumor cells were arranged in irregular nests, cords, and tubular and trabecular (Fig. 2A) patterns with a loose myxoid stroma (Fig. 2B). Focally, the tumor cells appeared to connect to the basal layer of the bronchial epithelium and lacked keratinization and in situ carcinoma of the overlying epithelium (Fig. 2C). Immunohistochemistry showed the tumor cells to be positive for CK7 (Fig. 2D); most tumor cells were found to be positive for p40 (Fig. 2E), ck5/6, p63 and negative for PAX8, CD10, RCC, CAIX, TTF1, S100, CDX2, SATB2, CK20, calponin, HMB-45, Melan A, SOX10, WT-1, Carteinin, D2–40, and SMA. An initial diagnosis of low-grade mucoepidermoid carcinoma with clear cell feathers was rendered. Because mucoepidermoid carcinoma of the lung is rare and involves specific molecular changes [12], molecular testing was performed. Fluorescence in situ hybridization (FISH) was negative for MAML2 gene rearrangement (Fig. 3A). These results prompted a histologic re-review of the case, a literature search, and consultation with an outside expert. The specialist, Dr. Zhou, found a resemblance between HCCC and myoepithelial tumors. Specifically, he found hyalinizing acellular stroma around the tumor cells in focal areas. Subsequently, additional FISH showed EWSR1 gene rearrangement, and EWSR1-ATF1 gene fusion confirmed the presence of the EWSR1 gene rearrangement (Fig. 3B) and EWSR1-ATF1 gene fusion (Fig. 3C). A final diagnosis of HCCC of the lung was ultimately rendered.

**Fig. 4:**
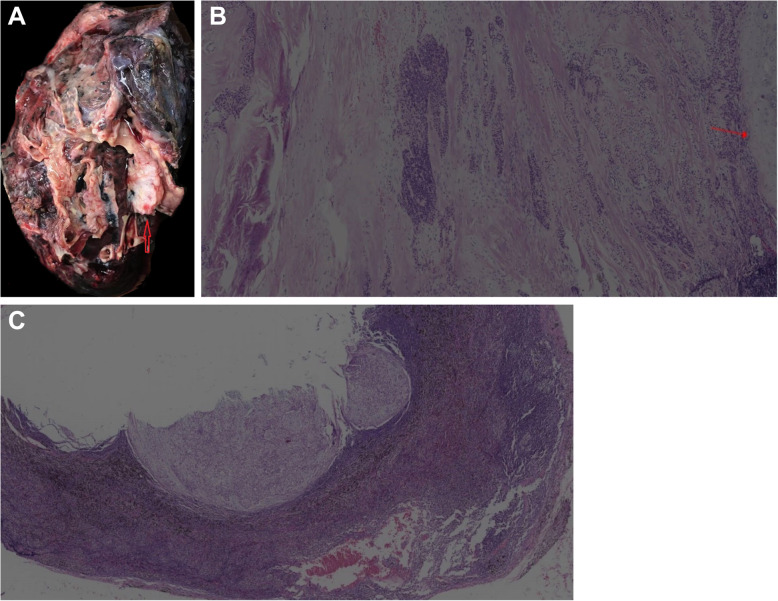
**A** The cut surface revealed a firm, solitary, well-circumscribed, 2.8-cm tan mass, 1.0 cm from the bronchial margin (red arrow). **B** Atypical small-to-medium sized epithelioid cells with light eosinophilic to clear cytoplasm embedded in a hyalinized stroma (the red arrow indicates bronchial cartilage). **C** The tumor cells that had metastasized to the lymph nodes showed the same morphology as the primary tumor

A right lower lobectomy with hilar and mediastinal lymphadenectomy was performed. Grossly, a firm, solitary, well-circumscribed, 2.8-cm tan mass, 1.0 cm from the bronchial margin, was identified (Fig. 4A). H&E-stained sections of the mass showed classical HCCC morphology: tumor cells with light eosinophilic to clear cytoplasm and inconspicuous nucleoli lacking significant pleomorphism and mitotic activity. The tumor cells grew in cords, trabeculae, and nests in a hyalinizing acellular stroma (Fig. 4B). They had infiltrated the periphery alveolar tissue and showed aggregates of chronic inflammation in the periphery of the tumor. No angiolymphatic invasion, perineural invasion, pleural involvement, or necrosis were observed. Immunohistochemistry and FISH results were the same as those of the biopsy. The metastatic foci were found in 1 out of 12 lymph nodes (Fig. 4C). All surgical margins were negative. At the time of this report, the patient was free of any local recurrence. She was free of metastasis at her 3-month follow-up, despite receiving no additional therapy after surgery.

In addition to the present case, 12 primary pulmonary HCCC cases that were previously reported in the English-language literature are summarized in Table 1 [4–11]. Of these 12 patients, 7 (60%) were women with an average age of 47.8 (range: 32–66 years), and 5 patients were smokers. These 12 patients, 5 cases presented with symptoms including cough, persistent back pain, and shortness of breath, and seven were asymptomatic. All primary tumors were located in the bronchial tubes or trachea. The average diameter of the tumor was 26.5 mm (range: 13–36 mm). Morphologically, every case showed nests or anastomosing cords of mixed clear and eosinophilic cells in a hyalinized or loose myxoid stroma. The tumor cells lacked significant pleomorphism and mitotic activity. Immunohistochemistry showed that the tumor cells were positive for CK7, P63, P40, and CK5/6, and negative for TTF-1, PAX-8, S-100, SMA, Napsin A, HMB45, Syn, and CgA.

**Table 1:** Summary of clinicopathologic features of all reported hyalinizing clear cell carcinomas of the lung

Patient	Size of tumor (mm)	IHC	Molecular Testing	Lymph node metastasis	Surgical Management	Follow-up Time/Outcome
38-year-old man, nonsmoker [4]	26	Positive:CK,CK7,P63,P40 negative:CK20,syn,CgA,S-100,SMA,TTF-1,Napsin A	Positive:EWSR1-ATF1 gene fusion, EWSR1 gene rearrangement negative:MAML2 gene rearrangement	N/A	Lobectomy	No recurrence or metastasis after 10 mo
32-year-old man, nonsmoker [5]	18	Positive:CK,CK7,P63 negative:CD10,CK20,PAX-8,syn,CgA,HMAB-45,S-100,SMA,TTF-1,Napsin A	Positive:EWSR1 gene rearrangement	N/A	Lobectomy	No recurrence or metastasis after 18 mo
39-year-old man, nonsmoker [5]	26	Positive:CK,CK7,P63 negative:CD10,CK20,PAX-8,syn,CgA,HMAB-45,S-100,SMA,TTF-1,Napsin A	Positive: EWSR1 gene rearrangement negative: MAML2 gene rearrangement	N/A	Lobectomy	No recurrence or metastasis after 18 mo
53-year-old man, nonsmoker [6]	N/A	Positive:CK,P63,P40 negative:CD10,CK20,PAX-8,syn,CgA,S-100,SMA,TTF-1,Napsin A,SOX10,CR	Positive: EWSR1 gene rearrangement,ATF1 gene rearrangement	N/A	Lobectomy	Lymph node metastasis/192 mo
54-year-old female, smoker [7]	35	Positive:CK,P63,CK5/6 negative:CK7,CK20,syn,CgA,TTF-1,Napsin A	Positive:EWSR1-ATF1 gene fusion	N/A	Lobectomy	No recurrence or metastasis after 16 mo
55-year-old man, smoker [8]	25	Positive:CK7,CK5/6,P63,P40 negative:TTF-1,Napsin A,CgA,Syn,CK20,S100,SMA	Positive:EWSR1 gene rearrangement	(-)	Lobectomy	No recurrence or metastasis after 20 mo
66-year-old female, smoker [9]	13	Positive:CK,CK7,CK5,p63 negative:TTF-1,Napsin A,CgA,Syn,S100	Positive:EWSR1 gene rearrangement	(-)	laser therapy	N/A
52-year-old female, nonsmoker [10]	33	Positive:CK7,CK5/6,P63,P40 negative:TTF-1,Napsin A,HMB45,Melan A,S100,CD1a,SOX10	Positive:EWSR1-ATF1 gene fusion, EWSR1 gene rearrangement	N/A	Lobectomy	No recurrence or metastasis after 181 mo
35-year-old female, smoker [10]	28	Positive:CK7,CK5/6,P63,P40 negative:TTF-1,Napsin A,HMB45,Melan A,S100,CD1a,SOX10	Positive:EWSR1-ATF1 gene fusion, EWSR1 gene rearrangement	N/A	Lobectomy	No recurrence or metastasis after 79 mo
56-year-old female, nonsmoker [10]	33	Positive:CK7,CK5/6,P63,P40 negative:TTF-1,Napsin A,HMB45,Melan A,S100,CD1a,SOX10	Positive:EWSR1-ATF1 gene fusion, EWSR1 gene rearrangement	(+)	Lobectomy	No recurrence or metastasis after 12 mo
46-year-old female, nonsmoker [11]	N/A	Positive:CK,P63 negative: S100,SMA	Positive:EWSR1-ATF1 gene fusion,negative: MAML2 gene rearrangement	N/A	Lobectomy	Deceased 6 years after first diagnosis
48-year-old female, smoker(current case)	28	Positive:CK7,CK5/6,P63,P40 negative:TTF-1,Napsin A,HMB45,Melan A,S100,WT-1,D2-40,SOX10	Positive:EWSR1-ATF1 gene fusion, EWSR1 gene rearrangement negative:MAML2 gene rearrangement	(+)	Lobectomy	No recurrence or metastasis after 3 mo

*N/A* Not applicable, *EWSR1* Ewing sarcoma breakpoint region 1, *ATF1* activating transcription factor 1, *MAML2* Mastermind-like 2

Molecular testing was done in each case, and showed that 10 cases were positive for EWSR1 gene rearrangement, seven cases were positive for EWSR1-ATF1 gene fusion, and four cases were negative for MAML2 gene rearrangement. Eleven cases were treated by surgical resection and one case by laser therapy. Lymph node metastasis was found in surgically removed specimens in only two cases. The average follow-up time was 56 months (with a range of 3 to 192 months), excluding case reported by Gubbiotti et al. [11] (that had recurrences over a 48-month period, and after which the patient became deceased 24 months after recurrence). Eleven cases of HCCC, when treated with complete surgical excision, exhibits an excellent prognosis with no evidence of mortality.

## Discussion

To our knowledge, only 11 cases of HCCC arising in the lung have been reported in the literature to date [4–11]. Three cases had biopsy results, and two of them first diagnosed by small biopsy were squamous cell carcinoma [7, 8]. Four of the 11 excised specimens were initially diagnosed as squamous cell carcinoma or mucoepidermoid carcinoma [4, 5, 11]; this indicated that the diagnosis of HCCC was difficult, especially from small biopsy samples. The small biopsy collected from our case showed that the tumor cells had undergone infiltrating growth and lacked significant pleomorphism and mitotic activity, indicating a low-grade carcinoma. Most tumor cells had light eosinophilic cytoplasm, and a small number of clear cells prompted us to consider several types of salivary gland-type tumors: low-grade mucoepidermoid carcinoma with clear cells [13], myoepithelial tumor [14], HCCC [4], metastatic clear cell renal cell carcinoma, or non-small-cell lung carcinoma. Immunohistochemistry result showed that the tumor cells were only positive for CK7, p40, ck5/6, and p63, which excluded metastatic clear cell renal cell carcinoma and lung adenocarcinoma. The mild cytological atypia and the lack of significant mitotic activity would have been unusual for the primary squamous cell carcinoma of the lung. Additionally, there was no evidence of squamous differentiation or in situ squamous carcinoma of the overlying epithelium; thus, we excluded squamous cell carcinoma. The initial diagnosis was low-grade mucoepidermoid carcinoma with prominent clear cells, given the homogeny of the cell population and the lack of mucous cells. Assessment of MAML2 by break-apart fluorescence in-situ hybridization, a highly sensitive and specific test for mucoepidermoid carcinoma, indicated that the tumor was intact. Based on these results, we excluded low-grade mucoepidermoid carcinoma.

Primary myoepithelial tumors in lungs are very rare [14]. They encompass benign myoepithelioma and malignant myoepithelial carcinoma. The distinction between myoepithelioma and myoepithelial carcinoma is largely based on morphologic features. Features that favor carcinoma include cytologic atypia, increased mitotic activity, necrosis, infiltrative growth, and metastatic disease [14, 15]. Myoepithelial tumors of salivary glands and HCCC were part of our diagnostic differentiations. They can have similar tumor cell morphology, growth pattern, and hyalinized, myxoid/hyalinized, or chondroid/hyalinized stroma [14]. Considering the infiltrative growth pattern of our case, we excluded benign myoepithelioma. Other morphologic data and the tumor stroma features indicated that it could be clear cell myoepithelial carcinoma or HCCC. However, myoepithelial carcinoma usually has more pleomorphism and, unlike HCCC, shows true myoepithelial differentiation with the expression of S100, SMA, and sometimes calponin. The EWSR1 gene rearrangement has been documented in both these types of cancers. Leckey Jr. et al. [16] reported a case of myoepithelial carcinoma of soft tissue with small round cell morphology having EWSR1-ATF1 gene fusion, but Skálová et al. [17] found that clear cell myoepithelial carcinoma with EWSR1 gene rearrangement frequently involved PLAG1 gene fusions but no EWSR1 fusion transcripts, while EWSR1-ATF1 gene fusion has been observed in most cases of HCCC [18]. We used FISH detection on the EWSR1-ATF1 chimeric gene. Results confirmed the presence of EWSR1-ATF1 gene fusion. Based on the tumor cell morphology and immunohistochemistry and FISH results, we confirmed the diagnosis of HCCC.

In primary tumors of the lung, EWSR1-ATF1 gene fusions have been well-characterized in clear cell sarcoma (CCS) [19], malignant mesothelioma (MM) [20], and angiomatoid fibrous histiocytoma (AFH) [21]. In our case, morphology and immunophenotype did not support the diagnosis of AFH. In order to rule out CCS and MM, additional immunohistochemical stains were performed and were negative for HMB-45, Melan A, SOX10, WT-1, Carteinin, and D2–40, not supporting these diagnoses either.

Because there have been few case reports of HCCCs metastasizing to cervical lymph nodes and the lung [22], we performed PET-CT evaluation. This confirmed the lungs to be the sole tumor site. Thus, we concluded that the tumor represented salivary-gland-type primary HCCC of the lung. The patient underwent surgery, and the excised specimens showed classical HCCC morphology. Immunohistochemistry and FISH characteristics were consistent with the diagnosis made based on this small biopsy.

A review of the literature found that 5 out of 12 cases were smokers. It was interesting to know the genomic signature of the HCCC showing smoking signatures or other signatures. Due to technical and specimen limitations, further research is required to find the genomic signature of primary lung HCCC.

In conclusion, it was extremely difficult to diagnose HCCC arising in the lungs by biopsy. It requires careful assessment of the morphology and adequate testing, such as immunohistochemistry and molecular testing.

## Data Availability

Data archiving is not mandated but data will be made available upon reasonable request.

## Abbreviations

HCCC: Hyalinizing clear cell carcinoma
PET-CT: Positron emission tomography scan
HE: Hematoxylin and eosin
FISH: Fluorescence in situ hybridization
